# Rapid Constructions of Microstructures for Optical Fiber Sensors Using a Commercial CO2 Laser System

**DOI:** 10.2174/1874120700802010028

**Published:** 2008-06-27

**Authors:** Rudi Irawan, Tjin Swee Chuan, Tay Chia Meng, Tan Khay Ming

**Affiliations:** 1 SUWA, BioMedical Engineering Research Centre, Nanyang Technological University, Singapore 637553; 2 Photonics Research Centre, School of EEE, Nanyang Technological University, Singapore 639798; 3 Department of Physics, University of Lampung, Bandar Lampung 35145, Indonesia

**Keywords:** Optical fiber, chemical or biological sensing, rapid construction of optical fiber sensors, fluorescence-based detection.

## Abstract

Exposing an optical fiber core to the measurand surrounding the fiber is often used to enhance the sensitivity of an optical fiber sensor. This paper reports on the rapid fabrication of microstructures in an optical fiber using a CO_2_ laser system which help exposing the optical fiber core to the measurand. The direct-write CO_2_ laser system used is originally designed for engraving the polymeric material. Fabrications of microstructures such as in-fiber microhole, D-shaped fiber, in-fiber microchannel, side-sliced fiber and tapered fiber were attempted. The microstructures in the fibers were examined using a SEM and an optical microscope. Quality of microstructures shown by the SEM images and promising results from fluorescence sensor tests using in-fiber microchannels of 100μm width, 210μm depth and 10mm length show the prospect of this method for use in optical fiber sensor development. The direct-write CO_2_ laser system is a flexible and fast machining tool for fabricating microstructures in an optical fiber, and can possibly be a replacement of the time consuming chemical etching and polishing methods used for microstructure fabrications of optical the fiber sensors reported in other literatures.

## INTRODUCTION 

The optical sensor is ubiquitous in today’s industry, existing to fill niches left by the electronic sensor, but in some cases it has taken over the electronic sensor as the mainstream sensor of choice due to its versatility. The optical sensor is best suited when measurements have to be taken in hazardous environments or when the sensor has to come into contact with the measurands or analytes [1]. Some examples of environments that degrade electronic sensors, but which optical sensors can survive in, are environments with high humidity, high temperature, high electromagnetic flux, and fluids. The fiber optic sensor is a class of optical sensors that has benefited from the boom in the fiber optic communications industry in the past twenty years. Major attributes of the fiber optic sensor are its dielectric property, intrinsic safety of a passive medium, immunity from electromagnetic interferences (EMI) and environmental noise, better sensitivity than many existing techniques, chemical inertness, light weight, small size and ease of installation afforded by its flexible geometry.

The most common fiber optic sensor and the easiest to demodulate are the intensity based sensor, which the intensity of light in the guided mode of the fiber is modulated by changes in the external environment [2]. The refractive index sensing and fluorescence sensing commonly use intensity based sensors. When a properly designed sensor reacts to changes in a physical quantity like refractive index or fluorescence intensity in the environment, the simple change of light intensity exiting a fiber sensor can possibly be correlated to the concentration of a measurand, which can be a biological or chemical analyte. Hence, this method is popularly used for chemical, biological and biochemical sensing. A fiber sensor can also be embedded in a microfluidic channel easily due to its flexibility, light weight and small size [3, 4]. The refractive index sensing has been used for detection of chemicals, or measurement of chemical concentrations in lab-on-chip or microfluidic applications [5-7]. The fluorescence based sensing is more commonly employed for biological analyte sensing and for analyte sorting in lab-on-a-chip applications due to its specificity and sensitivity [7-9].

When preparing a fiber for chemical and biological sensing, the light in the fiber core must be able to be modulated by the measurand surrounding the fiber. To achieve this objective, enhancing the interaction between the light guided in the fiber core and the measurand by exposing the fiber core to the external environment is popularly used. Microstructures are often needed in the fiber to promote light scattering as well as evanescent field coupling. The commonly used methods to obtain microstructures in the optical fiber sensor are side polishing of the fiber [5], etching the fiber to thin or to strip off the cladding [10], tapering the fiber tip [6] or shaping to D-shaped fiber [11, 12]. These present microstructure fabrication methods in fibers are time consuming, use expensive reagents, are less flexible to turn new fiber sensor designs and are therefore not practical for making large quantities or fast prototypes of sensors. Hence, a simpler and more effective fabrication technique is needed for the fabrication of microstructures in optical fiber sensors. Direct-write micromachining using CO_2_ lasers has been discussed with in-depth analysis and with a focus on creating microfluidic channels in polymethyl-methacrylate (PMMA) substrates [13-15] and on creating patterns in glass plates [16], but none on creating microstructures in optical fibers for optical fiber sensor applications.

In this paper, we report a direct write method for microfabrication of microstructures in optical fibers using a continuous wave (CW) CO_2_ laser system. It is believed that this technique can replace some of the conventional techniques, such as photomask technique, photolithography, etching processes and polishing processes which hamper the rapid turnaround of new fiber sensor designs [11]. The proposed method uses computer aided design software for drawing different patterns and designs which are transferred to the computer-controlled CO_2_ laser cutting machine which will then imprint the patterns directly onto the fiber. Hence, changing and improving designs are extremely rapid and simple, and making large quantities of sensors or prototyping new designs is fast as well. Different structures like D-shaped fiber, side-sliced fiber and tapered fiber tip as well as novel structures, in-fiber microholes and in-fiber microchannels, were fabricated, and the different types of fibers, single mode fibers, large core silica fibers and PMMA fibers were tested.

A fluorescence sensing application was tested in our experiments. The in-fiber microchannel structure, with a feature resolution of about 100μm, provides a novel geometry where biological or chemical fluids can flow and/or analytes can be immobilized within the in-fiber channel, and where the fluorescence sensing can take place. If the analytes of interest are fluorophore tagged analytes, this microstructure allows for efficient excitation and collection of fluorescence emission, which in turn improve the sensitivity of the sensor. As the analytes of interest are enclosed inside the structures within the fiber and the fluorescence emission is contained inside the structures, the fluorescence sensor using an in-fiber microchannel will provide good sensitivity. This fluorescence sensor will also be less sensitive to alignment and require a simpler optical set-up than the conventional fluorescence sensor does.

## IN-FIBER MICROSTRUCTURE FABRICATIONS

### Materials and Apparatus 

Three types of optical fibers were tested for microstructure fabrications. They were the PMMA optical fiber (PMMA fiber)- 750 μm core diameter with 10 µm fluorinated polymer cladding from Industrial Fiber Optics, Inc., large core silica fiber (PCS fiber)- 600μm/750μm (core/clad diameter) from Ceram Optec Industries Inc. and single mode optical fiber. A commercial direct-write CO_2_ laser machine, from Epilog Laser, was used to construct microstructures in fibers. It was operated at a wavelength of λ = 10.6 μm with full power of 40 W, which could be varied by the colors of computer drawings and by setting, and consisted of computer-controlled two-dimensional robotic arms with mirrors mounted at the arms to direct the laser beam across the entire machining area 61cm x 30.5 cm as depicted in Fig. (**1**). The microstructure patterns were designed and drawn using CorelDraw. A scanning electron microscope machine from JOEL was used to take images of constructed microstructures. The constructed microstructures were also visually observed using an optical microscope.

### Results and Discussion

Unlike UV-lasers, a CO_2_ laser creates the microstructures mainly due to the photothermal ablation mechanism. The far-infrared laser beam, at λ = 10.6 μm, is absorbed by fiber materials, then heats, melts, and vaporizes the materials. This process leaves a void in the work piece. If the laser beam is scanned along an optical fiber, microstructures will be created in the fiber. The patterns of microstructures are predetermined by the pattern drawings and the depth of microstructures are dictated by several factors, such as the characteristic of work pieces, power of laser beam, the number of laser passes and moving speed of laser scan. Careful control of laser intensity and number of laser passes determine the structure depth, but a balance must be adjusted between the laser power, number of laser passes and speed of laser scan to create well defined structures. The microstructure depth increases linearly with the laser power set [13]. However, the laser power needs to be optimized depending on the characteristics of materials. If too strong power is used, most of the surrounding material can be burned and the microstructures will not be well formed. The structure depth also increases linearly with the number of laser passes [13]. It was found that ideally lower power should be used, but the laser beam should be scanned multiple times over the same area of the optical fiber to create well defined structures. However, each laser source has its own minimum power and each material has its own minimum required power for ablation to be performed. It was found that the CO_2_ laser source used in this experiment sometimes fails to lase at power less than 10% of its maximum specified power. Because the glass transition temperature of silica is higher than PMMA, to obtain the same depth of structures the silica core fibers require higher laser power, or a greater number of laser passes, or both than PMMA fibers do. It was also found that the ablation process on the PMMA fibers caused the thin layer of white residue to be formed on the surface of microstructures. Annealing the fiber after fabrication at 80 ^o^C for an hour was able to eliminate the white residue partially. It was attempted to anneal the fiber at temperature 100 ^o^C or higher, but it deformed the microstructure and/or the fiber. On the other hand, the ablation process on the silica core fibers still maintained the microstructures with clear and relatively smooth surfaces. Any debris or particles were left by microfabrication processes on silica core fibers were easily cleaned by sonicating the microstructures in water.

The CO_2_ laser machine used was originally designed for engraving polymeric material sheets. However, in this experiment this laser set-up was tested to fabricate microstructures in optical fibers, both polymeric core optical fiber and silica core optical fiber, in a few seconds. This method does not only reduce the fabrication time, but also eases the complexity of development of an optical fiber sensor based on enhancing the fiber core’s exposure to measurand surrounding the fiber which is a common technique used for chemical and biological sensors. In order to fabricate different patterns of microstructures onto a piece of optical fiber, different writing patterns of CO_2_ laser must be used. These writing patterns can be predetermined easily by the designs drawn using a computer aided design software, such as CorelDraw, and then the designs are transferred to the computer-controlled CO_2_ laser machine to write the patterns on the work piece. The laser beam directed by mirrors mounted at the computer-controlled two-dimensional robotic arms illustrated in Fig. (**1**) writes the patterns onto the fibers.

Various patterns of microstructures, D-shaped fiber, in-fiber microhole, side sliced fiber, in-fiber microchannel and tapered fiber tip shown in Figs. ((**2**), (**3**), (**4**), (**5**) and (**6**)) were machined in this experiment. These patterns of microstructures were chosen because they are commonly used or have potential applications for optical fiber sensors. Fibers with microstructures, particularly PCS and single mode fibers, need to be handled gently and carefully, since they are very fragile.

The main feature of a D-shaped fiber is the flat surface on one side of a fiber as shown in Fig. (**2**). At the flat surface side, the core of optical fiber is exposed to or nearest to the measurand, and this, in turn, allows the light propagating inside the fiber to interact with the measurand through the evanescent wave or light scattering. A D-shaped optical fiber sensor is valuable for chemical, biological and biochemical sensing [11, 12, 17]. To achieve a nice D-shaped structure on a large core fiber, a pattern shown in Fig. (**7a**) needs to be prepared. The CO_2_ laser machine used recognizes different shades of gray in determination of laser intensity. The black color means that the incident laser power is maximized at the level set by the user and the white fill enables no writing on the work piece. Hence, a gray scale gradient enables a gradient of laser power incident on the work piece. The power gradient of the laser incident on an optical fiber is required due to the cylindrical shape of an optical fiber and the gradient depends on the size and materials of the fiber. To construct a D-shaped structure on a single mode fiber, a similar pattern can be used, but the laser scan must be vertical direction as illustrated in Fig. (**7b**). Scanning the laser a long single mode fiber possibly causes the whole fiber melted and vaporized, unless very low laser power is used and it is almost impossible to be achieved by using the laser source used in this experiment.

It is believed that a microhole structure in a fiber is one of the novel structures. The in-fiber microhole has a promising application for integrating the reaction chamber and fluorescence sensor [18], so that the sensing system is more versatile. The integration of the reaction chamber and sensor in a piece of fiber makes the system more compact, robust and sensitive. In fact, the pattern prepared to be written on a fiber as shown in Fig. (**7c**) was a step-well profile, a microhole with uniform depth. However, besides depending on the designed patterns, the cross section of a microhole depends on the thermal diffusivity of the material and on the intensity distribution within the CO_2_ laser beam. Since the thermal diffusivity of the optical fiber material used, silica core, is very low, it is believed that the intensity distribution in the laser beam dictates the microhole cross section. The intensity distribution of the CO_2_ laser beam had a Gaussian-like profile, so that the cross section of microholes obtained was a Gaussian-like profile as shown in Fig. (**3**).

The side sliced fiber has a depression like an elongated half cosine wave on its side as shown in Fig. (**4**). This type of microstructure was fabricated with a gradient profile as depicted in Fig. (**7d**). It was found that to get a better control on the profile, the direction of laser scan must be perpendicular to the fiber, particularly for a single mode fiber. With this laser scanning direction, a depression can be created nicely and smoothly on a fiber, with tapering side as seen in Fig. (**4**). The slopes and length of microstructure were determined by the gradient profile shown in Fig. (**7d**). The main advantage of a side sliced fiber sensor is it allows only sliced side of fiber to be exposed to the measurand, and the other side of the fiber still protected by its jacket and cladding may be buried into a substrate to save the brittle fiber sensor from mechanical forces [19]. The sliced side of fiber as biological or chemical sensing area can be faced into a microfluidic channel where the analytes flow or are treated, while the unsliced side can be buried inside the substrate of microfluidic chip to position the fiber.

In-fiber microchannel is another novel structure that has a promising application for improving the sensitivity and compactness of a fluorescence sensor. It will be discussed in more detail in the next section of this report. Together with sensing applications, the in-fiber microchannels in Figs. (**5**) may also be used as substrates of disposable microfluidic channels. These combined applications make in-fiber microchannel structures interesting for lab-on-chip applications, because an in-fiber microchannel structure improves not only the collection efficiency of fluorescence emission, but also robustness and compactness of the whole system. Originally the microchannel patterns prepared were step-channel profiles as illustrated in Fig. (**7e**). However, due to the same reasons as microholes’ fabrication, the cross-section of a microchannel obtained has a Gaussian-like profile as shown in Fig. (**8**). Fig. (**8**) also shows that the width of the microchannel surface was slightly bigger than 100 μm, the specified size, which might be due to that the position of the focal point of the laser system shifted from the channel surface as the channel became deeper. The channel surfaces in PCS fibers were optically clear by visual inspection and also confirmed *via *microscope observations. The SEM images and microscope observations showed that the channel bottoms were relatively smooth and no cracks. On the other hand, the ablation process on a PMMA fiber produced thin layer of white residue on the channel surface. As previously mentioned, this white residue can be partly eliminated by annealing a PMMA fiber after channel fabrication.

A tapered optical fiber tip is popularly used as a probe to detect or measure chemical or biological samples [6, 20]. Because of fiber flexibility and micro size, this structure is convenient for measuring or detecting inaccessible measurands. The fabrication of tapered fiber tips is somewhat similar to that of side polished fibers, but the structure is fabricated at the edge of the fiber as shown in Fig. (**7f**). Using the gradient pattern illustrated in Fig. (**7f**), the CO_2_ laser machine can write a ramp like structure at the edge of fiber, Fig. (**6**). For the same reasons as side polished fiber fabrication, the laser scan direction is suggested to be perpendicular to the fiber. It is possible to fabricate a circular tapered fiber tip, if the laser machine is equipped with a controlled device that can rotate a fiber uniformly and continuously.

This fabrication method has the potential for both fast flexible prototyping and reproducible mass production. To the best of our knowledge, no works so far have been published about the use of a direct-write CW CO_2_ laser machine to write microstructures in optical fibers for sensor applications. The conventional methods used for fabrication of microstructures in fibers are etching the silica fibers using chemical wet etching process or polishing the fibers that are time consuming processes that hamper the rapid turnaround of new fiber sensor designs. To realize a new fiber sensor design, the conventional methods may take one or two days, while the direct-write technique using CO_2_ laser machine requires only a few hours. Moreover, unlike the conventional methods, the direct-write technique using CO_2_ laser needs almost no consumables, so it is cheaper. The drawback of the direct-write CO_2_ laser technique is its resolution, since it uses long wavelength laser light, 10.6 µm. The smallest structure that can be constructed by the machine used in this experiment is about 80 µm.

## OPTICAL FIBER SENSOR APPLICATION TESTS 

### Methodology 

In-fiber microchannel structures, both in PMMA and silica core fibers, were chosen for sensor application tests in this experiment. The width, depth and length of the fabricated microchannels tested were approximately 100μm, 210 µm and 10 mm, respectively. To obtain this microchannel dimension, the power of CO_2_ laser machine was set at 32 W and 15 laser passes were given for fabrications on silica fibers. On the other hand, PMMA fibers were treated with the laser at 20 W and single laser pass. The SEM images of the in-fiber channel structures after fabrication are shown in Figs. (**5**) and (**8**).

Fluorescein (Sigma-Aldrich) was used as fluorophores for testing the optical fluorescence sensor. Fluorescein is widely available, inexpensive, and commonly used as a biomolecular fluorescence tag. Fluorescein powder was dissolved in phosphate-buffer saline (PBS) to stabilize its pH at 7.4, as its fluorescence spectrum is affected by pH of solution. The prepared fluorescein solutions were stored and enclosed properly to protect the solutions from excessive exposure to light before any measurement was conducted. Overexposing the fluorescein solution to light can lead to photobleaching. Different concentrations of fluorescein solutions from 0.001 μg/L to 10 g/L were prepared to characterize the response of the sensor.

Fig. (**9**) illustrates the experimental setup used in the testing of the fiber fluorescence sensor. The optical fiber with a microfluidic channel (the sensor section) under the test was installed inside an opaque black container. The both ends of the fiber were extended to the exterior of the container, so that the fiber can be accessed easily by the light source and detection systems. The light source used was a Nichia blue LED (NSPB300A, Nichia Chemical Industries) with an excitation wavelength centered at 470nm with a Full-Width Half-Maximum (FWHM) of about 28nm. A bandpass filter (SP Corion Filters, 470nm±5nm) was attached to the excitation source to limit the excitation spectrum, so that the excitation spectrum from the LED did not overlap with the fluorescence emission spectrum. The excitation light was focused into one end of the fiber and the fluorescence emission was collected at the other end of the fiber. The output of the fiber sensor was a mixture of the fluorescence and excitation light, so that a high pass filter (Andover Corp, 550nm highpass) was installed in front of the detector to filter out the excitation light from entering the detector. A 1 mm diameter pin hole was also attached to the detector to prevent stray light from entering the detector. Finally, the emission light was focused to a mini-compact module of photon multiplier tube (PMT, Hamamatsu H5784-02) which had the output connected to a volt meter.

To eliminate any possible errors due to some residue from previous measurements, the experiments were started from the lowest concentration of the prepared sample solutions, and then progressed to consecutively higher concentrations, although extra effort was made to clean the fibers thoroughly between the measurements. The container was filled with a known concentration of the fluorescein solution until the optical fiber was immersed inside the solution. Then, the solution was drained out, so that the microfluidic channel in the fiber was filled with solution, but with none remaining in the container. The same procedure was repeated for other measurements. The fiber and the container were cleaned thoroughly between the measurements by flushing deionized water through the container and in-fiber microchannel. Since a fluorescein solution is sensitive to photobleaching issue if exposed to light, to avoid pre-exposure of the fluorescein solutions, the shutter of the excitation light was only opened when the measurements of fluorescence emissions were performed.

### Results And Discussion 

The in-fiber microchannel structure was selected for sensor tests, because it is a novel structure and has potentially interesting applications in lab-on-chip. The in-fiber microchannel structure is interesting not only because it can be used for sensor applications, but also because it can be used as substrates of disposable microfluidic channels for lab-on-a-chip systems in which the fluorescence sensors are popularly used [8, 9, 21-25]. Its potential to be used both as the substrate of a disposable microfluidic channel and also as a fluorescence sensor will make a lab-on-a-chip system more compact, practical and robust. Conventional methods to measure fluorescence emissions from the samples in microchannels usually involve optically exciting the samples and detecting the fluorescence emissions from the surfaces of the channels. The excitation light is focused onto the sample and the fluorescence emission is collected and focused into a detector which can be at the same side as or the opposite side (with reference to the microchannel) from the excitation source. This type of setup usually hinders the compactness and simplicity of the system. Focusing a light beam onto a microchannel is challenging, particularly if the light beam diverges, such as light from LEDs used here. A poorly focused beam may cause the light to be scattered by the walls of microchannels, which in turn can be sources of noise and cross-talk [26]. Collection efficiency of fluorescence emission using this method is also low, usually less than 1% [27]. Moreover, this type of set-up is sensitive to the alignment issues pertaining to the focusing system, microchannel, and collection system. Any minor misalignment can cause severe problems to the consistency of the result. On the other hand, in the method using in-fiber microchannel tested in this experiment, the excitation light can be coupled more easily into the optical fiber, transmitted through the optical fiber and interacts with the fluorescence sample situated in the microchannel through evanescent wave and/or scattered light mechanisms. The evanescent and/or scattered light excites the fluorophores, and the isotropic fluorescence emission is collected efficiently by the same fiber that readily transmits the fluorescence light to the ends of a fiber sensor. The fluorescein solutions absorb the excitation light, blue light, and emit fluorescence, green light.

The fluorescence emission is isotropic in nature. Since the sample is contained inside the microchannel in the fiber, most of the fluorescence emission is coupled into the same fiber efficiently. The overall detection efficiency of this sensor system at one end of the fiber was estimated about 30%. Considering the simplicity of the optical system used, this detection efficiency is very good. In comparison with others, Schmidt *et al. *[28] and Khön *et al. *[29] achieved 3% and 10% efficiency respectively, but at the expense of complex optics.

Unlike conventional methods that usually take point measurements of fluorescence emission, the technique reported here collects fluorescence emission from along the channel. It may be necessary to have an averaged measurement over the whole channel, since immobilization processes of real biological samples in a microchannel are usually statistical processes. Hence, the fluorescence sensor based on the in-fiber microchannel adds at least a four-fold advantage to the sensor; namely providing the channel for sample detection, enhancing the exposure of the fiber core to the sample for light-matter interactions through evanescent wave and light scattering, averaging fluorescence emission over the whole channel and enhancing the coupling efficiency of fluorescence emission back into the fiber, which in turn increases the sensor sensitivity.

Despite the drawbacks of LEDs, such as diverging beams and broad band spectra, a blue LED was used as the excitation source. Because the LED was a relatively broad band source, a 470 nm interference band-pass filter needed to be installed at the light source. The output of the optical fiber sensor could be collected easily and focused into the detector, a mini-compact module PMT, as depicted in Fig. (**9**). This PMT module, which includes a high gain built-in amplifier and a built-in DC-DC high-voltage converter, is compact (2 cm x 2 cm x 6 cm) and operated only at 15 V DC. The use of this mini PMT module and a blue LED as an excitation source makes the system compact, cheap, simple and stable. A 550 nm wavelength of high pass filter was installed at the detector to suppress any vestiges of the excitation beam at the detection portion. A high pass filter of 500 nm can be sufficient, but this wavelength falls into the overlapping region of the excitation and emission spectra of fluorescein. In this region, some of the photons from the emission can be reabsorbed to excite fluoresceins. Therefore, it is better to exclude the photons from the overlap region that may create a complex relationship between fluorescence intensity and fluorescein concentration.

The system can be further miniaturized and simplified. The detection filter can be coated directly onto the detector window, and the blue LED can be replaced by a blue laser diode to eliminate the need of the excitation filter. Recently, a compact laser diode at 470 nm wavelength is becoming commercially available, but the cost remains expensive and the size is still relatively bulky. Otherwise, a laser diode at 470 nm wavelength is a good excitation source, as it has a very small line-width, less divergence beam and high intensity.

Figs. (**10a**) and (**b**)) demonstrate the promising results of both types of fiber sensors. The PCS fiber sensor and PMMA fiber sensor were able to detect lower than 0.05 μg/L and 0.5 μg/L fluorescein solutions respectively. The figures clearly show that the PCS fiber sensor gave better sensitivity and more linear response than the PMMA fiber sensor did. It might be due to a thin layer of white residue formed on the microchannel of PMMA fiber during the ablation process. It was observed under an optical microscope that the white residue was established at the walls of microchannels in PMMA fibers. This white thin layer might cause fouling effect in the channel that could block, absorb and scatter the fluorescence emission in the channel, which in turn degraded the sensitivity and the linearity of PMMA fiber sensor performance. This white residue was partly eliminated by annealing the PMMA fiber after fabrication to improve the sensor performance. On the other hand, the walls of microchannels in PCS fiber were relatively clear. No cracks were evident inside the microchannels. Scattering of fluorescence due to the surface roughness was more likely to degrade the PCS fiber sensor performance than the fouling effect. Although PMMA has very low background fluorescence [30], if background fluorescence is of concern, a PCS fiber should be used as silica theoretically has no background fluorescence. The results show the prospect of the in-fiber microchannel sensor used as a fluorescence sensor, such as a fluorescence immunosensor in a microfluidic chip.

## CONCLUSIONS 

This paper has reported the prospect of fabricating microstructures in optical fibers for sensor applications using a commercial CW CO_2_ laser system. It is believed that this technique can replace the conventional techniques, such as time consuming chemical etching and fiber polishing techniques, to construct an optical fiber sensor. Various microstructures in optical fibers, in-fiber microhole, D-shaped fiber, in-fiber microchannel, side-polished fiber and tapered fiber, were constructed easily and quickly. The commercial CO_2_ laser direct-writing machine, which was originally designed for engraving polymer materials, enables rapid prototyping and iterative design cycles of microstructures in both PMMA fiber and silica core fiber. The surface qualities of fabricated microstructures were relatively smooth and had no cracks. As designing and changing microstructure patterns are easy and fabrication process is rapid, this technique is very suitable for fast prototyping new sensor designs to test new ideas. The optical fiber is relatively inexpensive, so that the optical fiber sensor can be treated as a disposable and consumable component which is convenient for chemical, biological and biochemical sensing applications to avoid cross contaminations. The microfabrication using a CO_2_ laser machine may leave particles or debris in the structures that can be cleaned easily by sonicating the microstructures in water.

The fluorescence sensors using in-fiber microchannels were tested for both PCS fiber sensor and PMMA fiber sensor and they gave promising results. In-fiber microchannels of the PCS fiber sensor and the PMMA fiber sensor were able to detect fluorescein concentrations lower than 0.05 μg/L and 0.5 µg/L, respectively. Besides as the sensor area, the in-fiber channel can function as the microchannel for the flowing chemical or biological samples as well. The fluorescence sensor based on in-fiber channel also avoids complex geometrical optical systems to focus the excitation light into the microchannels and to collect the fluorescence emission from the samples inside the microchannels. These advantages make the in-fiber channel sensor is suitable for compact lab-on-chip applications. The results provide a platform to develop a compact, robust, sensitive and affordable fiber based optical sensor.

## Figures and Tables

**Fig. (1):**
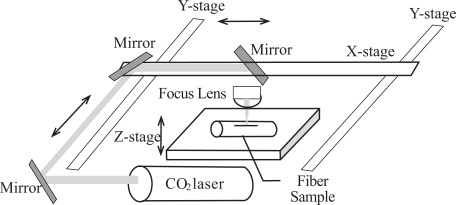
Layout of a direct write CO2 laser machine for rapid constructions of microstructures in fibers.

**Fig. (2):**
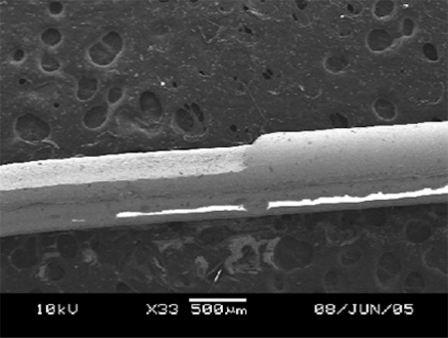
D-shaped fiber constructed on 600µm silica core fiber.

**Fig. (3):**
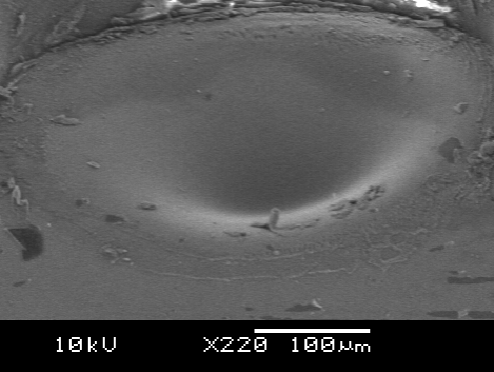
A microhole fabricated in a silica core fiber.

**Fig. (4):**
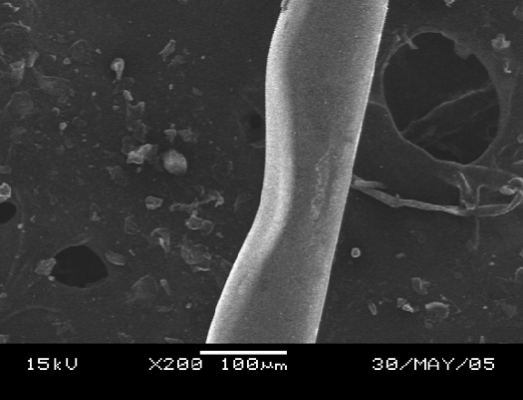
Side sliced single mode fiber.

**Fig. (5):**
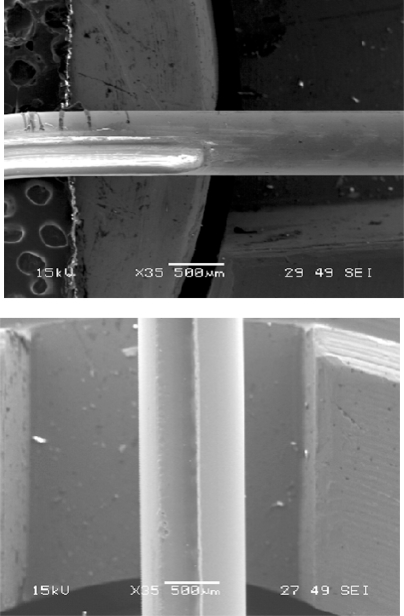
In-fiber microchannels written in (A) 600 µm silica core fiber, (B) PMMA fiber.

**Fig. (6):**
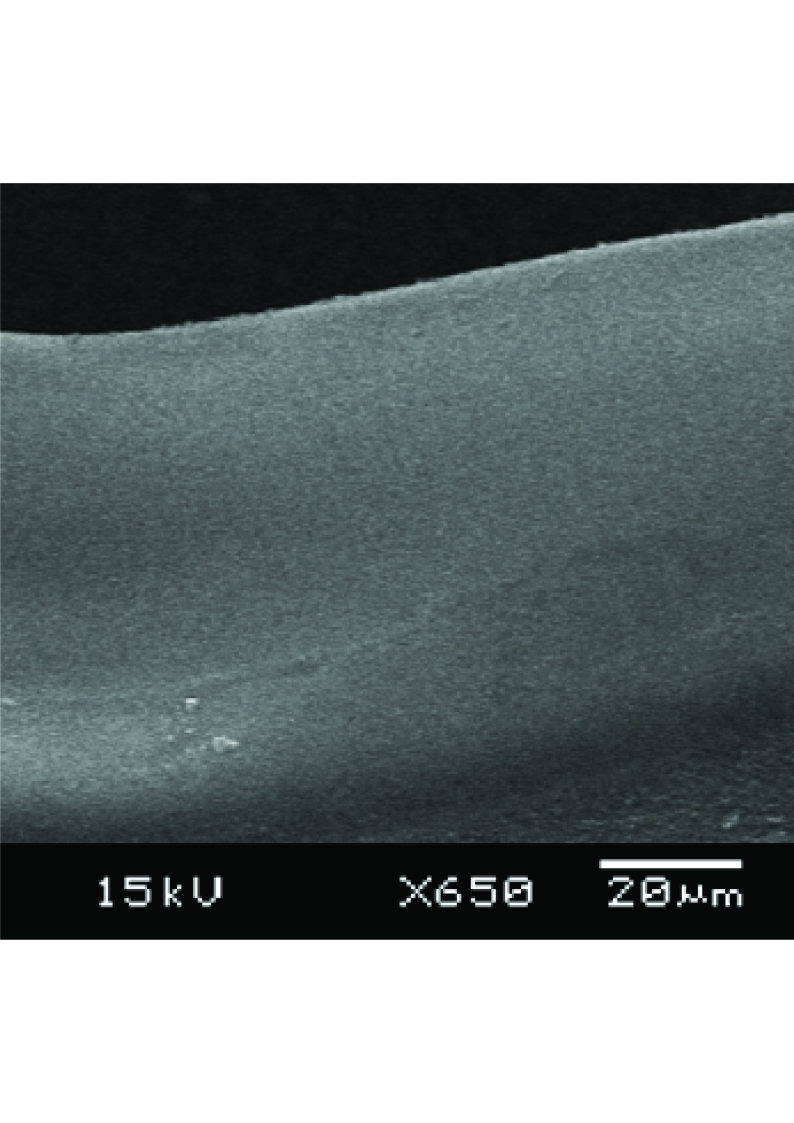
Tapered single mode fiber.

**Fig. (7):**
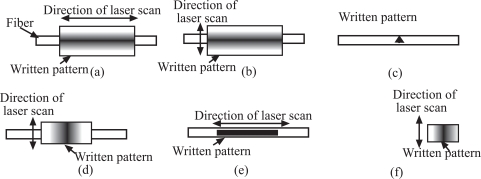
Patterns for fabrication of different microstructures in optical fibers using the CO2 laser machine. (a) D-shaped fiber for a large core fiber, (b) D-shaped fiber for a single mode fiber, (c) micro-hole, (d) side sliced fiber, (e) in-fiber microchannel and (f) tapered fiber tip.

**Fig. (8):**
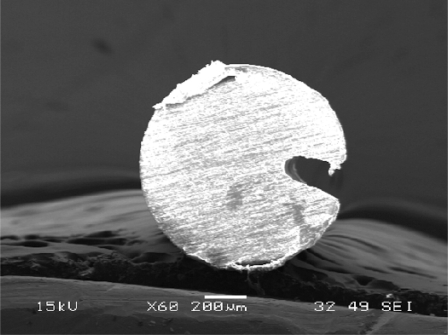
Cross section of in-fiber microchannel written in a PMMA fiber.

**Fig. (9):**
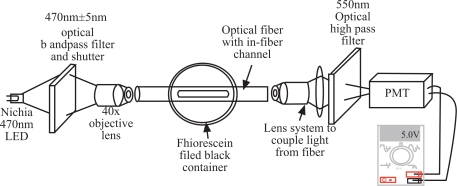
Experimental setup for testing of the fiber optic fluorescence sensor. PMT – photon multiplier tube.

**Fig. (10):**
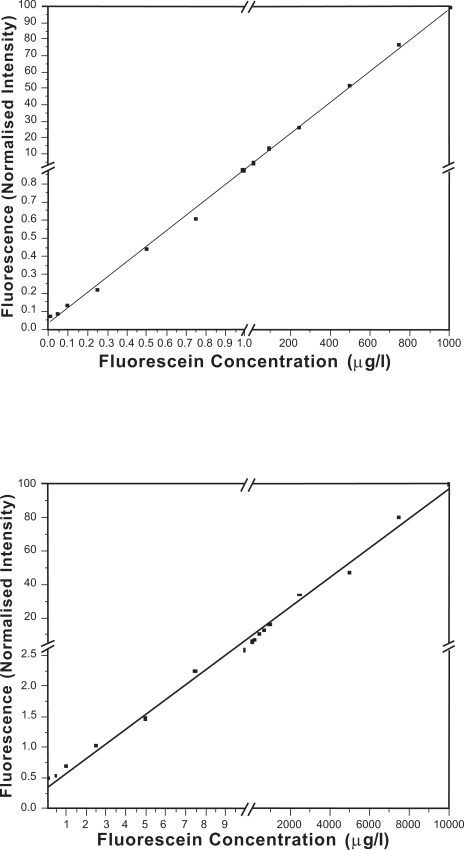
Concentrations of fluorescein in PBS pH 7.4 vs. fluorescence intensities: (a) in-fiber channel made from PCS fiber is used as an optical fiber sensor; (b) in-fiber channel made from PMMA fiber is used as an optical fiber sensor.
